# Gene Expression of *Haloferax volcanii* on Intermediate and Abundant Sources of Fixed Nitrogen

**DOI:** 10.3390/ijms20194784

**Published:** 2019-09-26

**Authors:** Sungmin Hwang, Nikita E. Chavarria, Rylee K. Hackley, Amy K. Schmid, Julie A. Maupin-Furlow

**Affiliations:** 1 Department of Biology, Duke University, Durham, NC 27708, USA; sungmin.hwang@duke.edu (S.H.); rylee.hackley@duke.edu (R.K.H.); 2 Department of Microbiology and Cell Science, Institute of Food and Agricultural Sciences, University of Florida, Gainesville, FL 32611, USA; nikita.chavarria@utrgv.edu; 3 University Program in Genetics and Genomics, Duke University, Durham, NC 27708, USA; 4 Center for Genomics and Computational Biology, Duke University, Durham, NC 27708, USA; 5 Genetics Institute, University of Florida, Gainesville, FL 32611, USA

**Keywords:** halophilic archaea, nitrogen metabolism, ammonium uptake, transcriptome, alanine transport, transcription factor, Lrp-type

## Abstract

*Haloferax volcanii*, a well-developed model archaeon for genomic, transcriptomic, and proteomic analyses, can grow on a defined medium of abundant and intermediate levels of fixed nitrogen. Here we report a global profiling of gene expression of *H. volcanii* grown on ammonium as an abundant source of fixed nitrogen compared to l-alanine, the latter of which exemplifies an intermediate source of nitrogen that can be obtained from dead cells in natural habitats. By comparing the two growth conditions, 30 genes were found to be differentially expressed, including 16 genes associated with amino acid metabolism and transport. The gene expression profiles contributed to mapping ammonium and l-alanine usage with respect to transporters and metabolic pathways. In addition, conserved DNA motifs were identified in the putative promoter regions and transcription factors were found to be in synteny with the differentially expressed genes, leading us to propose regulons of transcriptionally co-regulated operons. This study provides insight to how *H. volcanii* responds to and utilizes intermediate vs. abundant sources of fixed nitrogen for growth, with implications for conserved functions in related halophilic archaea.

## 1. Introduction

Transcription is a primary mechanism that enables organisms to regulate gene expression to produce cellular factors in response to changing environmental conditions. Traditional methodologies that detect differential gene expression, like Northern blots and quantitative reverse transcription-polymerase chain reaction (PCR) [1,2], are useful to characterize specific target genes. In concert, global transcriptional analyses using expression microarray, and most recently RNA-seq, are widely used to analyze the gene expression level of bacteria and eukaryotes in response to various stimuli and environmental conditions [3,4,5,6]. Transcriptomic analysis is also used as a pivotal tool to identify how archaeal gene expression is regulated under environmental stress or nutrient fluctuations [7,8,9,10].

Nitrogen, one of the most common elements on the earth, is present in different redox states from nitrate (NO_3_^−^) to ammonium (NH_4_^+^). The majority of nitrogen in biomolecules is present in reduced forms. Biologically, nitrogen comprises amide groups in amino acids and functional groups in nucleic acids, which are essential for growth and reproduction [11]. Interconversion of the different forms of nitrogen, termed N-cycle, is coordinated by biogeochemical redox processes. Nitrification, the oxidative conversion of ammonium into nitrite (NO_2_^−^) and further nitrate (NO_3_^−^), is mediated by a variety of bacteria and archaea in soils and oceans [12,13]. On the other hand, denitrifying microorganisms reduce NO_3_^−^ to nitrogenous compounds, such as NO_2_^−^, NO, N_2_O and N_2_ through denitrification processes [14]. N_2_ can be converted to ammonium by diazotrophs and archaeal methanogens through the assimilatory mechanism of nitrogen fixation; while ammonium is also generated from NO_3_^−^ and NO_2_^−^ by dissimilatory reduction through anaerobic respiration and by assimilatory reduction for cell carbon biosynthesis [15,16,17]. Considering that N-cycle compounds impact natural environments (e.g., N_2_O is a potent greenhouse gas) and human health (e.g., nitrate consumption is relevant to cancer and adverse reproductive outcomes), better understanding of nitrogen metabolism across diverse microbial groups will inform on the role of different environments in the global N-cycle [18,19]. 

Given the tremendous interest in understanding how archaea respond to and control nitrogen cycles, their transcriptional and post-transcriptional responses to nitrogen availability have been probed at the gene-specific and global levels. *Sulfolobus acidocaldarius*, a member of Crenarchaeota, encodes a leucine-responsive regulatory protein-like family transcriptional regulator Sa-Lrp [20]. In vitro Sa-Lrp binds specifically to the promoters of genes involved in nitrogen metabolism, such as glutamine synthetase (*glnA*-1, *glnA*-2, and *glnA*-3) and glutamate synthase (*gltB*) [20], suggesting that Sa-Lrp may regulate transcription of the GS/GOGAT pathway depending on N-signals. Transcriptional responses to N-conditions are best understood in the Euryarchaeota: the nitrogen-fixing methanogens [21,22] and the denitrifying halophile *Haloferax mediterranei* [8,23,24]. The methanogens use a master transcriptional repressor NrpR, secondary transcriptional activator NrpA, and small RNAs (sRNAs) to control the expression of glutamine synthetase (*glnA*) and nitrogenase (*nif*) genes in response to N-sources [22]. *H. mediterranei* mediates transcriptome level responses that are consistent with its capacity for denitrification. For instance, genes involved in the assimilatory reduction of nitrate to nitrite (*nasA*) and nitrite to ammonium (*nasD*) are up-regulated in the presence of nitrate [25,26]. In addition, the transcript levels of the high affinity Amt-type ammonia transporter and PII (GlnK) regulators are up during N-starvation in *H. mediterranei* [27]. The regulatory network responsible for controlling the N-responses in non-methanogenic haloarchaea, however, is poorly understood; sRNAs are implicated in control of the Amt/PII and glutamate dehydrogenase (*gdhA1*) genes [24], but a master NrpR or NrpA-type transcriptional regulator is not conserved. 

Here, we use global transcriptome profiling to examine the response of *Haloferax volcanii* to l-alanine vs. ammonium as a nitrogen (N-) source. *H. volcanii*, a close relative to *H. mediterranei*, is a model archaeon originally isolated from the Dead Sea (salinity ca. 340 g/L) [28], where N-sources are diverse and ammonia concentrations have gradually increased over time due to pollution [29]. l-alanine was chosen as the intermediate N-source based on prior knowledge that: i) *H. volcanii* can use l-alanine as an N-source [30]; and ii) the methanogenic archaeon *Methanococcus maripaludis* responds at the transcriptional level to l-alanine as an intermediate N-source [31]. 

Our findings provide insight into how halophilic archaea, such as *H. volcanii*, respond to intermediate (l-alanine) vs. abundant (ammonium) N-sources at the global level. Using microarray analysis, gene expression profiles were detected that enabled us to map putative transporters and metabolic pathways specific to the two N-sources. In synteny with these N-regulated operons, a putative *cis*-regulatory binding motif and an Lrp-like transcription factor were identified as candidates for controlling a transcriptional response to intermediate vs. abundant N-sources.

## 2. Results and Discussion

### 2.1. Genome-Wide Expression Analysis under Different Nitrogen Sources

**Figure A1:**
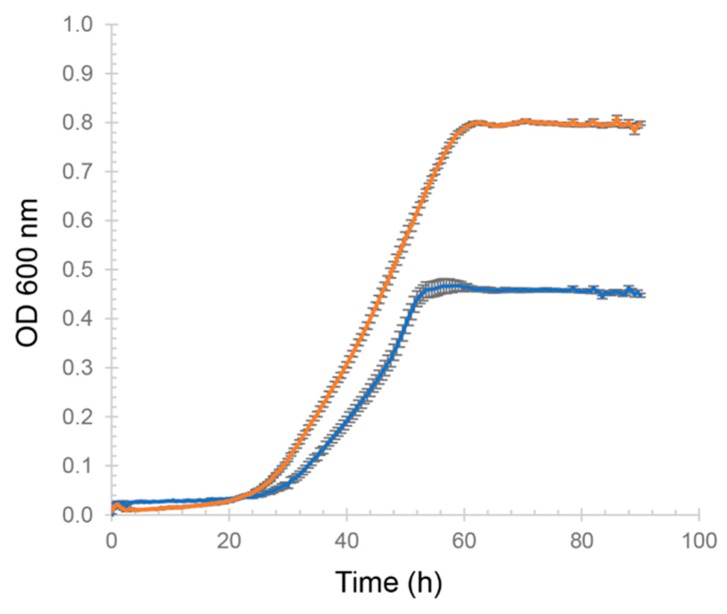
Growth of *H. volcanii* H26 in minimal medium with the N-source of ammonium (blue) and l-alanine (orange). Total *n* = 12 (biological quadruplicate and technical triplicate) was applied. Error bars, standard deviation of the mean. See Supplemental Methods in Appendix B for details.

*H. volcanii* H26 strain was analyzed for growth on glycerol minimal medium (GMM) with the N-source of 10 mM ammonium chloride (simplified to ‘ammonium or NH_4_^+^’) or 10 mM l-alanine (Figure A1). While the doubling time of H26 was similar under each condition (7.2 ± 0.14 h with l-alanine and 8.2 ± 0.19 h with ammonium; 1.1-fold difference), the final cell density in stationary phase (i.e., carrying capacity) increased 1.7-fold (*p* < 2.68 × 10^−15^, unpaired, two-sided *t*-test) when H26 was grown on l-alanine compared to ammonium (Figure A1). This distinction in cell density suggests that l-alanine may serve as a carbon source, in addition to serving as a N-source. The results also suggest that the cells may differ in their transcriptional response to these two N-sources. 

**Figure 1:**
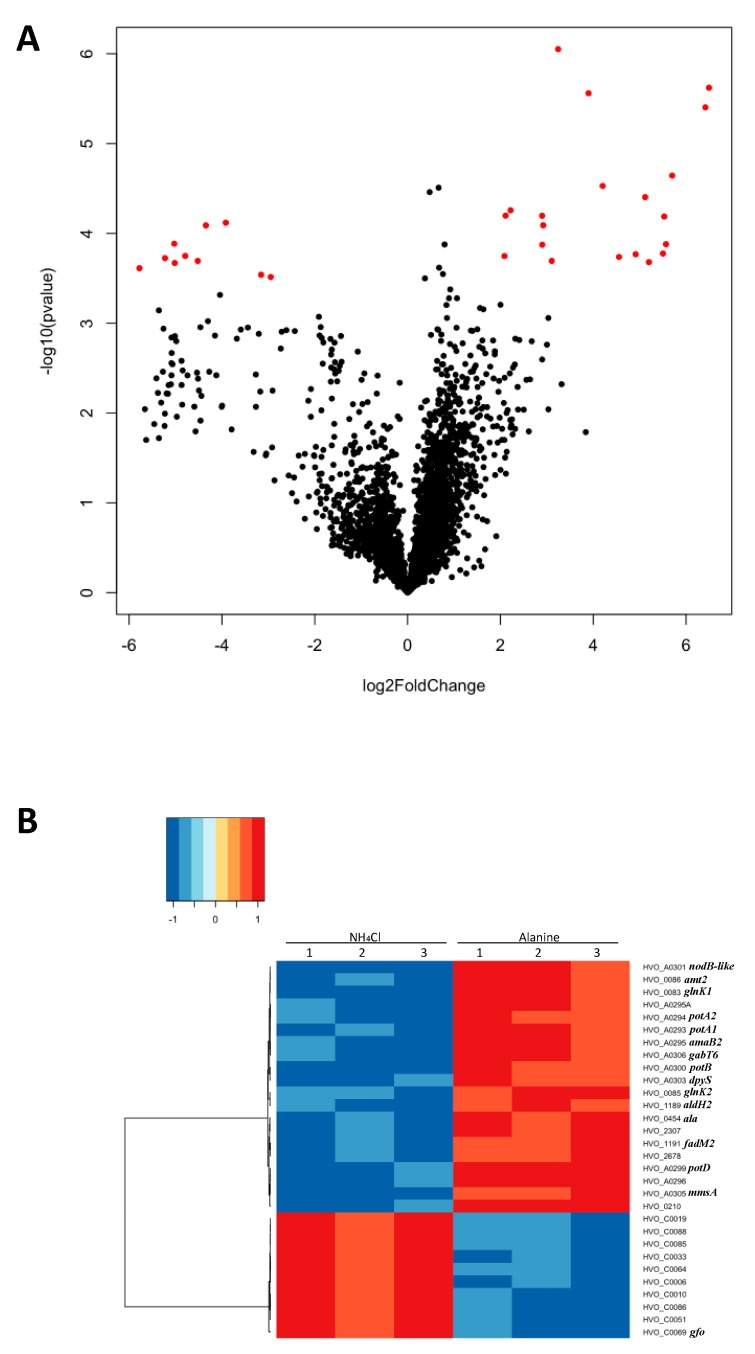
Microarray data analysis. (**A**) Each gene analyzed is visualized as a dot in the volcano plot. Red dots indicate genes whose log2-transformed expression ratio met the 2-fold and the false discovery rate (FDR) cutoffs. (**B**) The heat map represents the results of hierarchical clustering of genes differentially expressed in the growth conditions supplemented l-alanine compared to ammonium. Each gene in each row is labeled at the right side of the heat map with common gene names. Column numbers indicate the biological replicates under each condition. The dendrogram at the left shows the groups of co-expressed genes that resulted from clustering analysis. The color key at the top left indicates the correlation matrix (from −1 to 1 scale) of the log2 gene expression ratios in l-alanine: ammonium. See analysis details in Materials and Methods.

**Figure 2:**
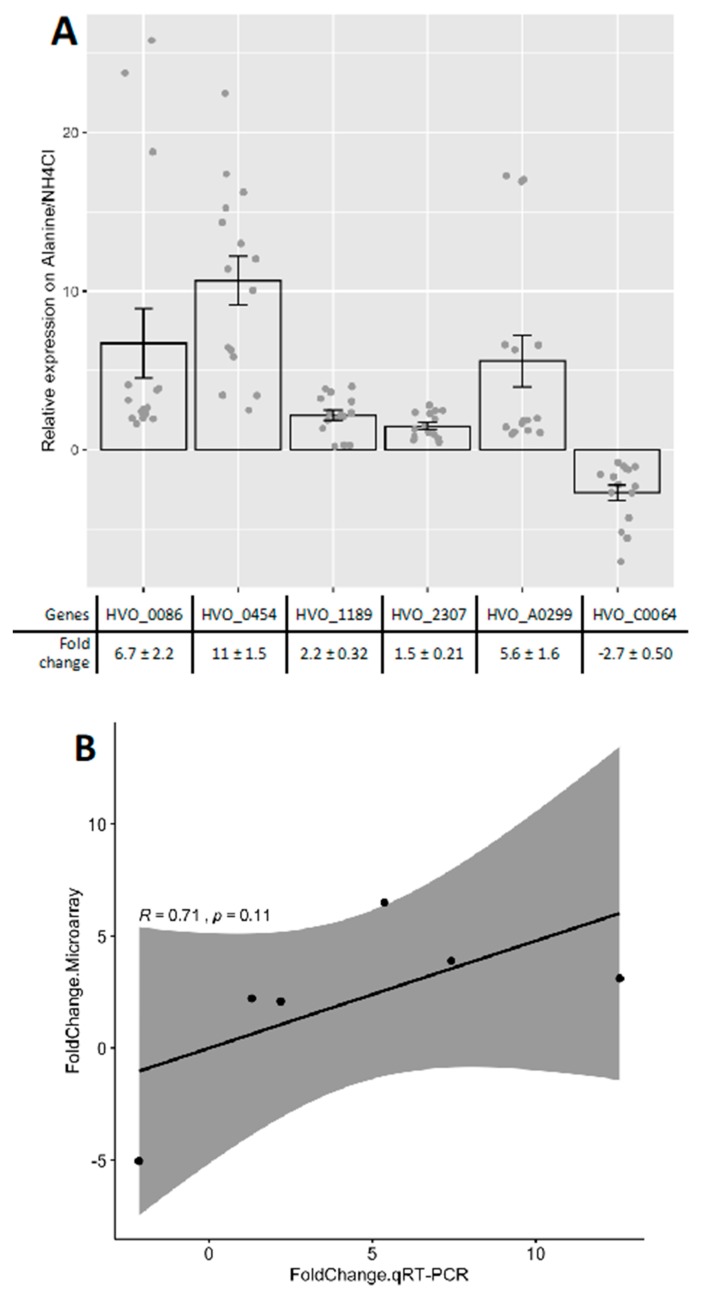
Validation of gene expression profiling by reverse transcriptional quantitative PCR (RT-qPCR). (**A**) Representative genes are from Table 1 and total *n* = 12 (biological quadruplicate and technical triplicate) was applied for the RT-qPCR. Error bars, standard error of the mean. (**B**) The expression magnitude of each genes between microarray and RT-qPCR was averaged and compared by Spearman’s correlation method.

To determine how cells respond to N-source shifts at the level of global gene expression, total RNA of H26 was isolated from log-phase cells grown with l-alanine or ammonium and the transcripts were analyzed by microarray. Expression was reproducible across biological replicates within each condition (correlation *R^2^* = 0.95–0.97 and 0.90–0.97 for ammonium and l-alanine, respectively, see also GitHub repository, URL given in Methods). In addition, average expression across all genes across the two conditions was *R^2^* = 0.90, suggesting good reproducibility of growth conditions with limited, condition-specific transcriptome changes. Differential expression was calculated for all genes by statistical analysis of the detected hybridization signals (Figure 1A, Methods). Thirty genes were identified as significantly differentially expressed between the two conditions, and those genes were clearly divided into two groups (Figure 1B): 10 genes were down-regulated and the remaining 20 genes were up-regulated in GMM with l-alanine versus ammonium (Table 1). These 30 differentially expressed genes were enriched for functions coding for amino acid metabolism and transport according to the eggNOG database for orthology mapping and functional classification [32] (*p* value ≤0.05, hypergeometric test). This functional enrichment of differentially expressed genes is in line with the biological role of nitrogen. RT-qPCR was performed to validate the genes identified by the microarray (Figure 2A). All the genes measured by RT-qPCR were up- or down-regulated on l-alanine/NH_4_Cl growth condition in concordance with the microarray data. Across all 6 genes measured, the two methods were strongly correlated (Spearman’s ρ = 0.71), with some gene-specific variation (Figure 2B). Taken together, these independent experimental results suggest that the genes identified by microarray are differentially expressed depending on the N-source.

**Figure A2:**
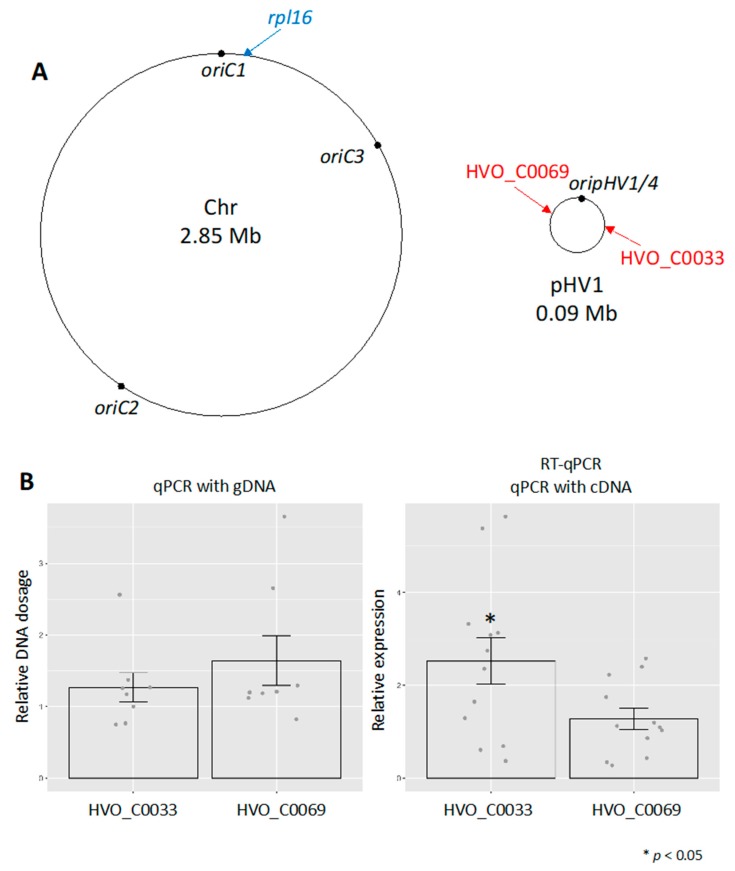
(**A**) Genomic locations of amplification targets for qPCR. Target genes in main chromosome and pHV1 are indicated in blue and red, respectively. Black dots show origin of replication in both chromosome and pHV1. (**B**) Relative copy number with gDNA (left) and cDNA (right) templates. Asterisks indicate *p* < 0.05 comparing the reference gene *rpl16* to sites of interest on pHV1 (See Supplemental Methods in Appendix B for details). Relative DNA dosage and mRNA gene expression (y-axes) are ratios of templates extracted from ammonium- vs. l-alanine-grown cells. Total n = 12 (biological quadruplicate and technical triplicate) and *n* = 8 (biological quadruplicate and technical duplicate) were applied to qPCR with cDNA and gDNA, respectively.

Notably, 8 of the 10 significantly down-regulated genes are predicted to encode hypothetical proteins with unknown function. The two exceptions are ISH4-type transposase and glucose-fructose oxidoreductase gene homologs (Table 1). All 10 genes down-regulated are encoded on plasmid pHV1. Surprisingly, the mean log2-fold value (alanine vs. ammonium) for all 75 genes encoded on pHV1 was −4.1 in this microarray analysis regardless of significance cutoff. In contrast, the mean expression for all genes on other genomic elements were: main chromosome 0.23; pHV2, 0.21; pHV3, −0.18; pHV4, 0.05. All the genes on pHV1 were down-regulated, whereas up- (66%) and down-regulated genes (34%) were evenly represented on the other chromosomal elements (Table S1). Gene expression level of one of the biological replicates in l-alanine (H26_ala_C in Table S1) differed from the other two with a *p* value < 2 × 10^−16^ (Pairwise Wilcoxon Rank Sum Test with Bonferroni Correction). However, the average log2-fold value (alanine vs. ammonium) of all pHV1 genes was still less than zero without this single biological replicate included in the average across biological replicates (H26_ala_C). Down-regulation of pHV1 genes was therefore not driven by differences between pHV1 expression across the biological replicates. One alternative explanation is that pHV1 gene expression was highly dependent on the replicon copy number and that N-source impacted this copy number through plasmid loss, rearrangement or altered polyploidy, as have been previously observed in *H. volcanii* and other halophiles [33,34,35]. To determine the relative copy number of the main chromosome and plasmid pHV1 in the two different growth conditions, quantitative PCR (qPCR) was performed with genomic DNA. The normalized amplicon level of two pHV1 genes, HVO_C0033 and HVO_C0069, was found to be similar to that of an internal control *rpl16* (HVO_0484) located on the main chromosome (Figure A2, Table S1). A previous study reported that the copy number of the main chromosome is 1.6-fold higher than that of pHV1 in exponential phase in *H. volcanii* [36]. The copy number discrepancy between the current and previous study might be caused by different growth conditions: complex vs. glycerol minimal medium. Further analysis of transcript levels by RT-qPCR showed HVO_C0033 and HVO_C0069 to be reduced in expression when cells were grown in l-alanine relative to ammonium (Figure A2B), in agreement with the microarray data. Taken together, these results suggest that the down-regulated gene expression in pHV1 during exposure to l-alanine vs. ammonium is not due to altered plasmid copy number. However, we cannot rule out that pHV1 was randomly lost during growth of cells for the microarray experiment but was maintained in cultures grown for the qPCR experiments (Figure A2B). The *H. volcanii* genome is highly plastic [35], and random gene loss on megaplasmids during microarray experiments has been observed in other halophiles with plastic genomes [33]. The mechanism involved in coordinated down-regulation across the entire pHV1 plasmid therefore remains unclear.

### 2.2. Putative Amino Acid Transport Systems were Upregulated on l-Alanine

**Figure 3:**
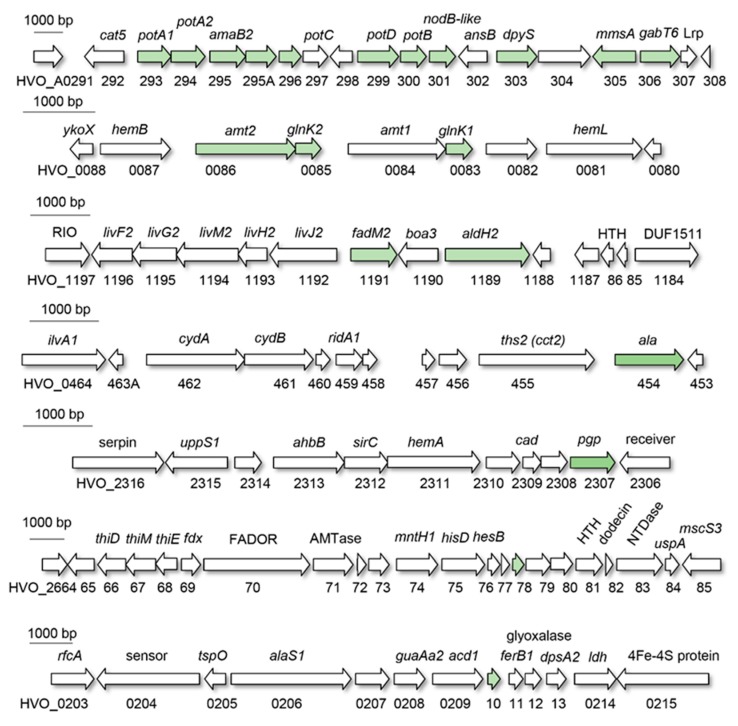
Operon organization of *H. volcanii* chromosomal and plasmid pHV4-based genes that are highly expressed on l-alanine compared to ammonium as an N-source. Arrows indicate open reading frames (ORFs) with those in green indicating genes that have transcripts more abundant on l-alanine compared to ammonium. Annotated gene names and locus tag numbers are indicated above and below the ORF, respectively.

**Figure 4:**
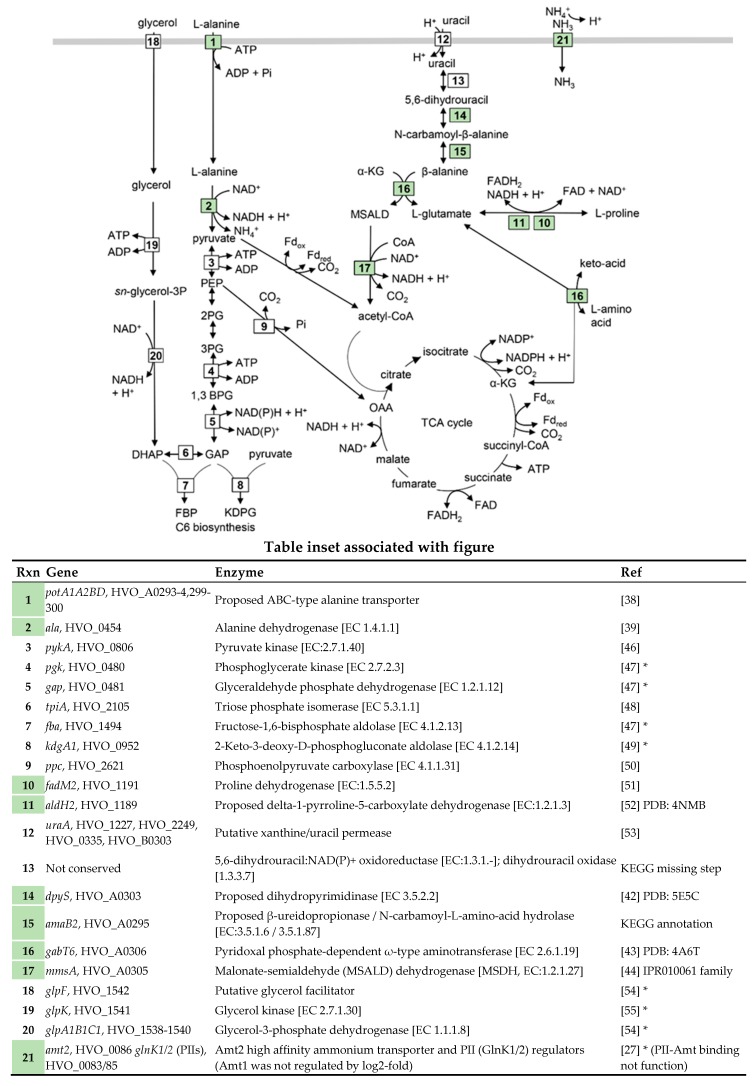
Model of metabolic and transport shifts that occur when glycerol grown *H. volcanii* cells use l-alanine instead of ammonium as an N-source. Reactions are defined in the Table inset with those highlighted in green associated with transcripts that are more abundant on l-alanine compared to ammonium. *, reference is specific for *Haloferax* sp. [46,47,48,49,50,51,52,53,54,55].

Of the 20 genes that were found upregulated at least 2-fold on l-alanine compared to ammonium as the N-source (Figure 3), seven were associated with transport (Figure 4). Included in this list were the Amt-type high affinity ammonium transporter (Amt2) and PII regulator (GlnK1 and GlnK2 or GlnK1/2) genes (Figure 3, Table 1). The PIIs are predicted to regulate transport of ammonium by Amt2 based on analogy to *E. coli* [37]. *H. mediterranei* undergoes a similar increase in *amt-glnK* transcript abundance when starved for nitrogen (N) [27]. Thus, in halophilic archaea, the Amt-transport system is upregulated by shifts to intermediate sources of fixed nitrogen in addition to N-limitation presumably to scavenge ammonium from the environment. The other transport system upregulated on l-alanine was the ABC-type transporter system PotA1, PotA2, PotB and PotD (PotA1A2BD) (Figure 4). While annotated as a potential spermidine/putrescine transporter (UniProt, April 10, 2019 update), the PotB permease of this system had a MetI-like transmembrane domain (IPR000515) related to the D-methionine ABC transporter of *E. coli* [38]. The high expression of *potA1A2BD* on l-alanine (*potB* and *potD* were among the most highly upregulated transcripts, Table 1), combined with the relationship of PotB to MetI, suggest a function of the encoded ABC-type system in the transport of amino acids such as l-alanine.

### 2.3. Upregulation of Metabolic Systems

Seven of the 20 genes upregulated on l-alanine compared to ammonium were mapped to metabolic pathways. Of these upregulated genes, HVO_0454 (*ala*) shares 46% identity and 60% similarity in amino acid sequence to the l-alanine dehydrogenase (AlaDH) of *Archaeoglobus fulgidus* that catalyzes the NAD^+^-dependent deamination of l-alanine to ammonium and pyruvate [39]. Thus, HVO_0454 (*ala*) likely catalyzes the first step of l-alanine metabolism (Figure 4) to generate ammonium as a source of fixed nitrogen, as well as pyruvate and reduced cofactor for the biosynthesis of cell carbon and energy.

Based on the transcript profiles, growth on GMM with l-alanine as the N-source also appears to stimulate the production of l-glutamate and acetyl-CoA through uracil and α-ketoglutarate metabolism (Figure 4 and table inset). l-glutamate would be beneficial as an amino-group donor for transaminases that convert α-keto acids to l-amino acids [40], while acetyl-CoA could serve as a substrate for the TCA cycle and an acetyl-group donor for other metabolic reactions [41]. Uracil and α-ketoglutarate are predicted to be converted to l-glutamate and acetyl-CoA during growth on l-alanine based on the following. The transcript levels of the gene neighbors HVO_A0295 (*amaB2*), HVO_A0303 (*dpyS*), HVO_A0305 (*mmsA*), and HVO_A0306 (*gabT6*) were up during grow on l-alanine vs. ammonium. Of these gene products, DpyS and AmaB2 are predicted to convert 5,6-dihydrouracil (an intermediate of uracil metabolism) to β-alanine; DpyS is modeled to be a 3D-structural homolog of a bacterial dihydropyrimidinase [42] and AmaB2 is classified as a β-ureidopropionase/ *N*-carbamoyl-l-amino-acid hydrolase in the KEGG database. GabT6 is suggested to transfer the amino group from β-alanine to α-ketoglutarate, as it is modeled to be a 3D-structural homolog of ω-type aminotransferases (that transfer virtually any primary amino group to various ketones [43]) and is divergently transcribed from the (methyl)malonate-semialdehyde dehydrogenase gene homolog *mmsA* [44]. GabT6 would thus form l-glutamate and malonate-semialdehyde (MSALD), the latter which may be oxidatively decarboxylated by MmsA to generate NADH and acetyl-CoA [44]. The source of uracil to feed into this β-alanine metabolic network may originate from the uracil supplement used to compensate for the *∆pyrE2* (orotate phosphoribosyl transferase) mutation of the model strain *H. volcanii* H26 [45]. This type of model strain is commonly used in archaeal genetics as a ‘wild type’ to allow for targeted gene deletion by homologous recombination through uracil selection and 5-fluoroorotic acid (FOA) counterselection.

In addition to l-alanine and β-alanine metabolism, genes predicted to function in the oxidation of l-proline to l-glutamate were also upregulated (Figure 4). Included in this list were HVO_1191 (*fadM2* or *putA*), a homolog of the archaeal-type proline dehydrogenase (ProDH) [51], and its gene neighbor HVO_1189 (*aldH2*) which shares predicted 3D structural homology to delta-1-pyrroline-5-carboxylate dehydrogenase (P5CDH) (PDB: 4NMB) [52]. ProDH and P5CDH are enzymes well characterized for their concerted action in converting l-proline to l-glutamate [51,52]. The metabolic signal that would alter the expression of the ProDH and P5CDH gene homologs is unclear. Enhanced levels of l-proline are not predicted to occur in l-alanine vs. ammonium grown cells. Instead, the cells appear to be responding to ammonium limitation based on the enhanced expression of Amt/PII system components on l-alanine vs. ammonium. Thus, the signal for upregulation of ProDH/P5CDH gene homologs could be a general response to ammonium limitation, as the metabolic product of these enzymes, l-glutamate, is central to N-metabolism.

### 2.4. Identification of a Candidate Regulator and cis-Sequence for Coordinated Transcriptional Control

Genes whose transcript abundance increased significantly on l-alanine compared to ammonium as an N-source clustered into seven distinct regions on the *H. volcanii* genome (Figure 3). The most striking was finding that 11 of the highly expressed genes (HVO_A0293 to HVO_A0306) spanned an 18.6 kb region of plasmid pHV4 at position CP001955.1: 303,472..322,061. The other regions of note were located on the main chromosome, including the *amt2-glnK2 amt1-glnK1* operons at as well as the ProDH (*fadM2*, HVO_1191) and P5CDH (*aldH2*, HVO_1189) homologs at position CP001956.1: 83,382..87,266 and 1,080,889..1,084,135, respectively. Based on this gene clustering, we hypothesized that common promoter elements may coordinate transcriptional responses to the type of N-source.

**Figure 5:**
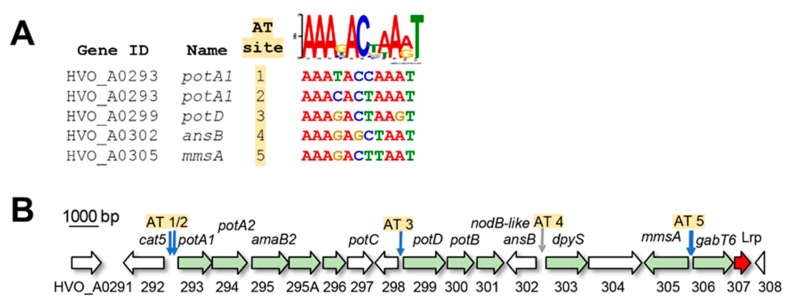
Putative cis-regulatory sequence motif and Lrp-like transcriptional regulator located in the 11 gene N-transport and metabolism cluster. (**A**) Motif logo in upper right corner depicts the position weight matrix results from the computational motif search. Genes containing the motif are listed at left, with motif sequences identified by genome scanning (FIMO) listed at right under the motif logo. AT-rich site numbers correspond to genomic locations within the gene cluster shown in (B). (**B**) Genomic location of motifs (vertical arrows) relative to differentially expressed ORFs (green horizontal arrows) within the HVO_A0291 – HVO_A0307 cluster. Putative Lrp-like transcriptional regulator is indicated by the red arrow. Blue vertical arrows indicate high confidence motifs, and the grey arrow indicates a low confidence motif (see also Table S2 and Figure A3).

To identify putative DNA binding motifs that may regulate genes linked to N-shifts, *de novo* motif identification searches were performed (see Materials and Methods for details). Briefly, genes associated with or regulated by the l-alanine N-shift were scanned using MEME for a common motif (input sequences included 5′ regions of *potA1*, *amaB2*, *potD*, *dpyS*, *gabT6*, *ala*, *amt2*, *amt1*, and *lrp*, Table S2). An AT-rich motif with best fit to a semi-palindromic 11 bp sequence AAAGACTAART was identified by this analysis. Using the FIMO program to search the *H. volcanii* genome, this motif was detected in the upstream regions of 492 genes (Table S2), with high statistical support for the motif consensus in regions 5′ of genes differentially expressed in response to l-alanine (*p* < 0.0027, Wilcoxon test vs. randomized sequences, see Methods, Figure 5A, and Table 1). Compared to the rest of the genome, the set of differentially expressed genes was enriched for the motif (hypergeometric test *p* < 3.22 × 10^−6^, Table S2). Eleven of the 30 differentially expressed genes were located within the 16-gene cluster involved in N-transport and metabolism, with the high confidence motifs located upstream of *potA1*, *potD*, *mmsA,* and *gabT6* (all highly expressed on l-alanine) (Figure 2A and Figure 5B, Table 1).

**Figure 6:**
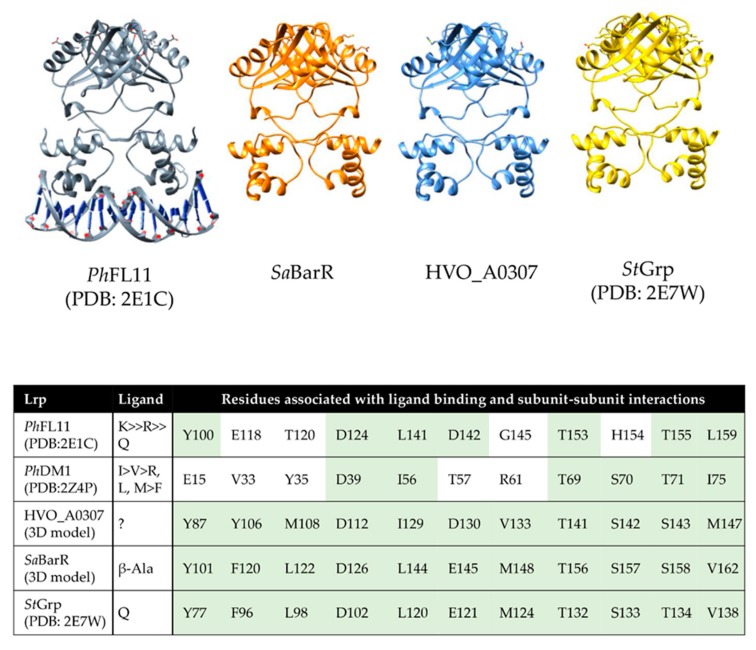
Full-length FFRP subgroup member HVO_A0307 and its relationship to other Lrp-type regulators. Upper: X-ray crystal structures of *Ph*FL11 (PDB: 2E1C) bound to DNA and *St*Grp (PDB: 2E7W) compared to 3D models of *Sa*BarR (Saci_2136) and HVO_A0307. When compared to all X-ray crystal structures available in the PDB databases (August 30, 2019) by homology modeling, HVO_A0307 was most closely related to *St*Grp (Lrp-type glutamine receptor protein) (PDB: 2E7W) at an root mean square deviation (RMSD) between 134 pruned atom pairs of 0.576 Å; (across all 149 pairs: 1.234 Å). The 3D models of HVO_A0307 and *Sa*BarR have an RMSD between 140 pruned atom pairs of 0.240 Å; (across all 148 pairs: 0.884 Å). Table inset: Residues of Lrp-type proteins found at the positions at which *Ph*DM1 interacts with ligand and/or alters subunit interactions [58]. Identical and similar residues to HVO_A0307 are highlighted in green. Abbreviations: Ph, *Pyrococcus horikoshii*; Sa, *Sulfolobus acidocaldarius*; St, *Sulfolobus tokodaii*.

Of the four genes identified to have high confidence AT-rich motifs, *mmsA* and *gabT6* were found to be in genome synteny with the transcription factor homolog HVO_A0307 (*lrp*) (Figure 3 and Figure 5). This type of genomic organization is observed in other archaea [56]. Of particular note is the *Sulfolobus* BarR (Saci_2136 and ST1115) Lrp-type transcription factor that shares 32% identity with HVO_0307 (*lrp*) and is genetically linked to *mmsA* and/or *gabT6* gene homologs [56,57], including the *Sulfolobus* ST1116 that is 45% identical to *mmsA* and the *Sulfolobus* ω-type aminotransferases Saci_2137 and ST1114 that are 43%-44% identical to *gabT6*. The *Sulfolobus* BarR binds a semi-palindromic AT-rich motif that repeats evenly in an intergenic region 5′ of the divergently transcribed *barR* and the *gabT6*-like gene [56]. Similarly to *Sulfolobus* BarR, HVO_A0307 is of the full-length feast/famine regulatory protein (FFRP) subgroup of the Lrp family transcriptional regulators that have an N-terminal DNA binding domain and a C-terminal domain that promotes self-assembly [58]. When compared to the X-ray crystal structures in the PDB database (August 30, 2019) by Phyre2-based structural homology modeling, HVO_A0307 and BarR were found to be most closely related to the *Sulfolobus tokodaii St*Grp (Lrp-type glutamine receptor protein) (PDB: 2E7W) (Figure 6). Further analysis revealed HVO_A0307 (*lrp*) to have conserved amino acid residues with BarR and *St*Grp at positions proposed to interact with ligand and/or influence self-assembly of the transcription factor [58]. Thus, HVO_A0307 is proposed to be an Lrp-type transcriptional regulator that may bind the repetitive AT-rich motifs 5′ of *potA1*, *potD*, *mmsA*, and *gabT6* (Figure 5B) in response to metabolic intermediates that signal growth on l-alanine vs. ammonium as the N-source (e.g., l-alanine, β-alanine, l-glutamine, and/or l-glutamate). The genes with this 5′ AT-rich motif (*potA1*, *potD*, *mmsA*, and *gabT6*) are in apparent operons with the l-alanine induced *potA2*, *amaB2*, *hvo_a0295a*, *hvo_a0295*, *hvo_a0296*, *potB,* and *hvo_a0301,* consistent with the hypothesis that the entire l-alanine-induced pHV4 region is regulated by the Lrp-related HVO_A0307.

## 3. Materials and Methods

### 3.1. Growth of H. volcanii

*H. volcanii* H26 was grown at 42 °C with shaking (200 rpm) in ATCC 974 complex medium (Hv-CM) or glycerol minimal medium (GMM). GMM was as previously described [45,59] with 20 mM glycerol as the main carbon source and 10 mM ammonium chloride (NH_4_Cl) or l-alanine as the N-source; uracil (50 μg∙ml^−1^) was included in GMM to allow for growth of H26 *∆pyrE2* [45,59]. The GMM formula per liter was: 20 mM glycerol, 141 g NaCl, 17.6 g MgCl_2_, 20.6 g MgSO_4_, 4.12 g KCl, 432 mg CaCl_2_, 0.353 mg MnCl_2_, 0.432 mg ZnSO_4_, 2.26 mg FeSO_4_, 0.0471 mg CuSO_4_, 50 mg uracil, 0.882 mg biotin, 0.882 mg thiamine, 1.91 mL of 0.5 M KPO_4_ buffer (pH7.5), and 35.3 mL of 1 M Tris-Cl (pH7.5) buffer with 10 mM NH_4_Cl or l-alanine as the variable.

A previous method was used to monitor growth rates under these conditions [60]. Briefly, single colonies of H26 were first inoculated in 5-mL Hv-CM and grown aerobically at 42 °C to early stationary phase (OD_600nm_, ~1.0). Cells were harvested via centrifugation (15,871× *g*, 1 min at room temperature) and washed two times with GMM without added N-sources. Cultures were then diluted to a starting OD_600nm_ of 0.03 in 200 μL of GMM with 10 mM NH_4_Cl or l-alanine under continuous shaking at 42 °C in a Bioscreen C analysis system (Growth Curves USA, Piscataway, NJ, USA) set to measure OD_600nm_ every 30 min. Each condition was tested using four independent biological replicate samples, each with three technical replicates.

### 3.2. Preparation and Analysis of Microarray Data

H26 was grown aerobically to exponential phase (OD_600nm_, 0.3 to 0.5) in GMM with two different N-sources (10 mM), NH_4_Cl or l-alanine. Total RNA preparation, NimbleGen microarray slides, cDNA synthesis and dye hybridization were as previously reported [61]. Double-stranded cDNA libraries were generated using the Superscript cDNA synthesis kit (Invitrogen, Carlsbad, CA, USA) following the manufacturer’s instructions. One microgram of the library from each biological replicate was labeled with Cy3 dye and hybridized to NimbleGen 12 × 135-k feature single-color custom microarray slides as described in the kit (Roche NimbleGen, Inc., Madison, WI, USA), with each 135-k array containing 98% of the annotated genes in the *H. volcanii* genome (3,985 genes, NCBI Genome ID, 1149) [61]. Microarray hybridization and scanning were conducted at the FSU-NimbleGen-certified facility (The Florida State University, Tallahassee, FL, USA). For each gene, 96 replicate data points were measured (32 replicate probes per gene per array, with 3 biological replicate hybridizations per sample). Raw spot intensities were first normalized within arrays using RMA, followed by normalization and analysis using the Subio Platform v. 1.22 (https://www.subioplatform.com) with the following parameters: filtering out signals below the low signal cutoff (raw intensity <1.0), global normalization (75^th^ percentile) and log2 transformation. Subsequent statistical tests, plotting, and analysis of microarray data were conducted R statistical computing environment (http://www.R-project.org). Resultant expression intensities (Table S1) were averaged across the four replicate probes for each gene in each growth condition, then subject to Student’s *t* test followed by Benjamini-Hochberg correction for multiple hypothesis testing [62]. The *q* value (false discovery rate, FDR) <0.05 was set as a cutoff, yielding the final list of 30 genes in Table 1. To generate heatmaps, genes were clustered by gene expression pattern across the three biological replicates using average linkage hierarchical clustering and plotted using the ggplot2 package [63]. Significance of enrichment of the differentially expressed genes by functional categories was determined using the hypergeometric test with Benjamini-Hochberg correction. Annotations were computed using eggNOG-mapper [32] based on eggNOG 4.5 orthology data. eggNOG is a public database of orthologous groups across different taxonomic levels and leverages several databases and text mining to call functional predictions. All code used in this study can be found in the GitHub repository: https://github.com/sungminhwang-duke/Microarray_N_sources. The microarray platform used in this study is available through NCBI Gene Expression Omnibus (GEO) at accession number GPL21414, and the raw and processed microarray data are available at accession number GSE130934.

### 3.3. Computational Prediction of Transcription Factor Binding Sequences

Computational prediction of DNA motifs was performed as follows. DNA sequences in the 5′ direction of selected genes differentially expressed in response to nitrogen or within operons of differentially expressed genes (*potA1*, *amaB2*, *potD*, *dpyS*, *gabT6*, *ala*, *amt2*, *amt1*, and *lrp*, in Table S2) were retrieved using the graphics tool within the NCBI nucleotide portal (https://www.ncbi.nlm.nih.gov/nuccore/). De novo motif detection was performed with these primary DNA sequences as input using the MEME Suite version 5.0.5 [64]. The parameters were set to any number of repeats, maximum width of 14-17 bp, reverse complement motifs allowed, and 3 output motifs. The FIMO algorithm from the MEME Suite was used to scan the *H. volcanii* genome (uid46845 June 11,2018 version within the Upstream Sequences: Prokaryotic database) for additional instances of the putative motifs using default parameters. Sequences containing the motif most often associated with differentially expressed genes were shuffled and compared to original sequences using the Wilcoxon signed-rank test to determine significance. Shuffling was conducted to preserve dinucleotide frequencies using the fasta-shuffle-letters command within the MEME suite (the underlying algorithm was based on uShuffle [65]). Details regarding the DNA motif analysis (input sequences, FIMO output) are given in Table S2.

## 4. Conclusions

Global transcript profiling of *H. volcanii* grown on a C3 source of glycerol with abundant (ammonium) verses intermediate (l-alanine) sources of fixed nitrogen revealed a distinct set of genes that were regulated including 20 genes that were upregulated and 10 genes that were downregulated on l-alanine. Of the upregulated genes, the majority could be mapped to the transport (7 genes) and metabolism (7 genes) of ammonium, l-alanine, and/or other associated metabolites. The transport systems included the high affinity ammonium transporter homolog Amt2 and its PII regulators GlnK1/2 as well as the ABC-type PotA1A2BD system which is suggested to transport amino acids such as l-alanine. Based on the metabolic genes that were upregulated, the oxidative deamination of l-alanine to pyruvate appeared to be of central importance to the l-alanine grown cells. Gene neighbors encoding an apparent pathway to synthesize l-glutamate and acetyl-CoA from uracil and α-ketoglutarate also were enhanced at the level of transcript abundance under the l-alanine conditions. Genome clustering of the upregulated genes was observed. This clustering enabled us to generate a model that the Lrp-type HVO_A0307 transcription factor homolog is a candidate for regulating the transcription of genes associated with N-shifts from ammonium to l-alanine. Thus, we propose that transcriptional co-regulation of a syntenic cluster of operons enables *H. volcanii* to respond to and utilize intermediate vs. abundant sources of fixed nitrogen for growth. 

## Figures and Tables

**Table 1:** Genes differential expression on l-alanine compared to ammonium in minimal medium.

Uniprot Annotation ^1^; Proposed Function ^2^	Gene	Log2 Fold Change	SD ^3^	SD Error
ABC-type transport system periplasmic substrate-binding protein (probable substrate spermidine/putrescine); l-alanine transport	HVO_A0299, *potD* ^5^	6.495	0.177	0.102
Polysaccharide deacetylase family protein	HVO_A0301, *nodB*-like	6.416	0.280	0.161
(Methyl)malonate-semialdehyde dehydrogenase [EC:1.2.1.27]	HVO_A0305, *mmsA* ^5^	5.701	0.310	0.179
Luciferase family protein; oxidoreductase	HVO_A0295A	5.569	0.396	0.228
ABC-type transport system permease protein (probable substrate spermidine/putrescine); l-alanine transport	HVO_A0300, *potB* ^4^	5.530	0.609	0.351
Amidase (hydantoinase/carbamoylase family); β-ureidopropionase / *N*-carbamoyl-l-amino-acid hydrolase [EC:3.5.1.6/3.5.1.87]	HVO_A0295, *amaB2* ^4^	5.504	0.637	0.368
Putative allantoinase; dihydropyrimidinase [EC 3.5.2.2]	HVO_A0303, *dpyS* ^5^	5.200	0.771	0.445
ABC-type transport system ATP-binding protein (probable substrate spermidine/putrescine); l-alanine transport	HVO_A0294, *potA2* ^4^	5.119	0.293	0.169
ABC-type transport system ATP-binding protein (probable substrate spermidine/putrescine); l-alanine transport	HVO_A0293, *potA1* ^4,5^	4.913	0.656	0.379
Pyridoxal phosphate-dependent aminotransferase	HVO_A0306, *gabT6* ^4,5^	4.555	0.508	0.293
Nitrogen regulatory protein P-II	HVO_0085, *glnK2* ^4^	4.203	0.241	0.139
Transport protein (Probable substrate ammonium)	HVO_0086, *amt2*	3.898	0.165	0.095
Short-chain family oxidoreductase	HVO_A0296	3.244	0.106	0.061
Alanine dehydrogenase [EC 1.4.1.1]	HVO_0454, *ala* ^4^	3.107	0.537	0.31
Nitrogen regulatory protein P-II	HVO_0083, *glnK1* ^4,5^	2.922	0.303	0.175
Proline dehydrogenase [EC:1.5.5.2]	HVO_1191, *fadM2* ^4^	2.901	0.280	0.161
Uncharacterized protein (arCOG09242)	HVO_0210	2.898	0.335	0.193
HAD superfamily hydrolase with phosphoglycolate phosphatase-like, domain 2 (IPR023198)	HVO_2307	2.22	0.270	0.156
Uncharacterized protein of arCOG06214	HVO_2678	2.113	0.231	0.133
Aldehyde dehydrogenase; delta-1-pyrroline-5-carboxylate dehydrogenase [EC:1.2.1.3]	HVO_1189, *aldH2*	2.086	0.243	0.140
Glucose-fructose oxidoreductase	HVO_C0069, *gfo*	−2.949	0.167	0.096
HTH domain protein	HVO_C0051 ^5^	−3.156	0.414	0.239
Uncharacterized protein	HVO_C0010	−3.918	0.376	0.217
Uncharacterized protein	HVO_C0033	−4.347	0.541	0.313
Small CPxCG-related zinc finger protein	HVO_C0086	−4.522	0.381	0.220
UPF0395 family protein	HVO_C0006	−4.792	0.715	0.413
Homolog to virus protein eHPD7_00180	HVO_C0085	−5.018	0.585	0.338
ISH4-type transposase ISHvo5	HVO_C0064	−5.027	0.662	0.382
hypothetical protein	HVO_C0019	−5.227	0.778	0.449
hypothetical protein	HVO_C0088	−5.779	0.837	0.483

^1^ Uniprot annotation as of May 14, 2019.; ^2^ Proposed by this study; ^3^ SD, standard deviation of the log2FoldChange value; ^4^ Genes of the enriched eggNOG category of amino acid metabolism; ^5^ Promoter contains predicted DNA binding motif.

**Figure A3:**
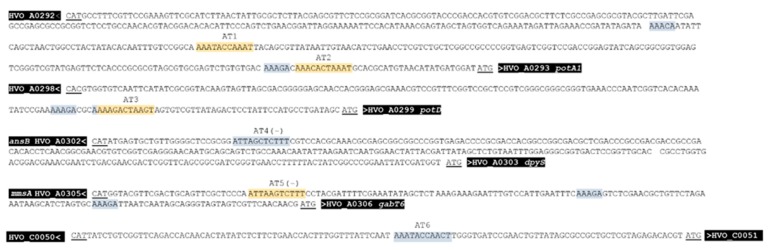
Sequences of putative regulatory motifs detected upstream of genes differentially expressed during a N-shift from ammonium to l-alanine (see also Figure 5 in main text). See Materials and Methods for motif detection details. AT-rich sites detected by the FIMO program with *p*-value < 1 × 10^−7^ are shown in yellow (high confidence sites labelled AT1, 2, 3, 5) and those with *p*-value > 1 × 10^−7^ in blue (low confidence sites labelled AT4, 6). AAAGA repeats outside of the AT-rich motif are highlighted in blue. Start codons are underlined. (–) represents DNA motif of complementary DNA sequence.

## Supplementary Materials

Supplementary materials can be found at https://www.mdpi.com/1422-0067/20/19/4784/s1.

## Appendix B. Supplemental Methods

### Appendix B.1. Growth Assay

Growth assay was as previously described [60]. Briefly, single colonies of H26 were inoculated in 5-mL Hv-CA (casamino acid) and grown aerobically at 42 °C to early stationary phase to synchronize growth phase (OD_600nm_, ~1.0). Cells were harvested via centrifugation (15,871× *g*, 1 min at room temperature) and washed two times with minimal medium without added N-sources. Cultures were diluted to a starting OD_600nm_ of 0.03 in 200 μL of minimal medium containing uracil with 10 mM NH_4_Cl or l-alanine under continuous shaking at 42 °C in a Bioscreen C analysis system (Growth Curves USA, Piscataway, NJ, USA) set to measure every 30 min at OD_600nm_. Each condition was tested using four biological samples, each with three technical replicates, as presented in Figure A1.

### Appendix B.2. Quantitative PCR to Calculate Copy Number and Gene Expression

Genomic DNA (gDNA) was extracted from 3 mL of cell culture as described by Dulmage et al. [33]. In brief, four biological replicate H26 were grown in minimal medium (see main text methods for details on media formulations), including uracil (50 μg·mL^−1^), with 10 mM l-alanine or 10 mM NH_4_Cl. Cells were harvested at OD_600nm_ ~ 0.5 by centrifugation (15,871× *g*, 30 s). The cell pellet was added to 250 µL of water and 250 µL of lysis buffer with the recipe described in *The Halohandbook* [59] and incubated for 5 min at room temperature (RT) with an occasional vortex. RNA was removed by RNase A (100 µg, 5 min at RT) and protein was degraded by Proteinase K (100 µg) at 37 °C for 10 min. Cell lysates were mixed with an equal volume of phenol/chloroform/ isoamyl alcohol (25 : 24 : 1) and DNA was precipitated in ethanol and resuspended in TE buffer. In addition, total RNA was isolated by using an Absolute RNA Miniprep kit (Agilent Technologies, Santa Clara, CA, USA) according to the procedures provided in the kit. An additional DNA digestion step was applied by using a Turbo DNA-free kit (Invitrogen, Carlsbad, CA, USA). The level of DNA contamination after the DNase treatment was checked by 35-cycles of end-point PCR with primers (HVO0863external_FW and HVO0863external_RV) targeting up/downstream of HVO_0863 (see primers in Table A1). For quantification, ten nanograms of gDNA or total RNA per reaction mixture volume (10 µL) served as the template. Quantitative PCR (qPCR) was carried out by qPCRBIO SyGreen 1-Step Go Lo-R kit (PCR Biosystems Inc., Wayne, PA, USA). One-step qPCR was performed under conditions of 45 °C for 10 min, 95 °C for 2 min, and 40 cycles of 95 °C for 15 s and 60 °C for 30 s, followed by determination of the melting curve by using a LightCycler 96 (Roche, Madison, WI, USA). The expression level of HVO_C0033 and HVO_C0069 were normalized to the internal gene, *rpl16*. The relative gene expression in ammonium-grown vs. l-alanine-grown cells was calculated by Livak’s method. Oligonucleotides used for qPCR (Table A1) were quantified for efficiency, which was determined to be between 90 and 110 percent. Gene expression for each target gene was compared to the internal *rpl16* gene to calculate significance by Welch’s *t*-test, and the correlation of the two independent experiments, microarray and RT-qPCR, was calculated by Spearman’s method.

**Table A1:** Oligonucleotides for quantitative PCR in this study.

Name	Sequence (5′-3′)	Description	Ref.
HVO0863external_FW	TCCCGGAGAGGTGTGTATGT	To test DNA contamination. Targeting up/downstream of HVO_0863. Amplicon: 1,767 bp.	This study
HVO0863external_RV	TCACTGACCTCGAACACGTC	To test DNA contamination. Targeting up/downstream of HVO_0863. Amplicon: 1,767 bp.	This study
rpl16s1_F	CCACGTCATCCGCGAGAACA	Control locus in main chromosome. Amplicon: 86 bp.	[66]
rpl16s1_R	CGACCTTCCCGAACGACTGG	Control locus in main chromosome. Amplicon: 86 bp.	[66]
qHVO_C0033_s2_F	CTCGGTTCGACAGGCCAAGG	Target locus in pHV1. Amplicon: 113 bp.	This study
qHVO_C0033_s2_R	CAGTTCCGCACATGGCATCG	Target locus in pHV1. Amplicon: 113 bp.	This study
qHVO_C0069_s2_F	AGGCACCGACTGTCTCGTCA	Target locus in pHV1. Amplicon: 124 bp.	This study
qHVO_C0069_s2_R	GAAGGAGAATCGCGCGGTGA	Target locus in pHV1. Amplicon: 124 bp.	This study
qHVO_A0299_s2_F	ACCGCCTACGGAACCTCAGT	Target locus in pHV4. Amplicon: 126 bp.	This study
qHVO_A0299_s2_R	CTCGGACATCGTGGCGTAGG	Target locus in pHV4. Amplicon: 126 bp.	This study
qHVO_0454_s2_F	CGGAGCTCATCTCGGCCATC	Target locus in main chromosome. Amplicon: 92 bp.	This study
qHVO_0454_s2_R	CTGCGGCAGGTCGATGTAGG	Target locus in main chromosome. Amplicon: 92 bp.	This study
qHVO_0086_s1_F	CGCGTGGATTGACTGGCTCT	Target locus in main chromosome. Amplicon: 100 bp.	This study
qHVO_0086_s1_R	GATGTACGCGCGGAAGTCCA	Target locus in main chromosome. Amplicon: 100 bp.	This study
qHVO_1189_s1_F	CTCCGAGGACTACCGCCAGA	Target locus in main chromosome. Amplicon: 81 bp.	This study
qHVO_1189_s1_R	CCGATACACGGGAGGTTGCC	Target locus in main chromosome. Amplicon: 81 bp.	This study
qHVO_2307_s1_F	TTCGACCTCGACGGAACCCT	Target locus in main chromosome. Amplicon: 127 bp.	This study
qHVO_2307_s1_R	CGAGTCCGTGCCAGATGGTC	Target locus in main chromosome. Amplicon: 127 bp.	This study
qHVO_C0064_s1_F	GCGAATACGTCGTCCACGGT	Target locus in pHV1. Amplicon: 70 bp.	This study
qHVO_C0064_s1_R	TGGCTCTCGCAGCTGTTGAC	Target locus in pHV1. Amplicon: 70 bp.	This study
